# Resignation

**Published:** 1856-08

**Authors:** 


"*Resignation.* — In February last, Dr. E. H. Parker, Professor of Anatomy in the New York Medical College, sent in his resignation of the chair occupied by him in that institution. Other engagements, inconsistent with the duties of a public teacher, and somewhat unexpectedly devolved upon him, made this step necessary. The resignation has not yet been accepted, but as it is insisted upon by Dr. Parker, it undoubtedly will be and a successor soon appointed."

